# Giant Liver Hemangiomas: A Plea for Early Surgical Referral and Resection

**DOI:** 10.1155/2020/5923787

**Published:** 2020-06-16

**Authors:** Alvin Chang, Brianna Ruch, Aamir Khan, Marlon Levy, Amit Sharma

**Affiliations:** School of Medicine and Hume-Lee Transplant Center, Virginia Commonwealth University, Richmond, Virginia 23298, USA

## Abstract

Hepatic hemangiomas are the most common benign hepatic tumor. Current guidelines recommend surveillance imaging and reserving surgical intervention for symptomatic patients with giant liver hemangiomas (>5 cm). We present the case of a patient with a rapidly enlarging giant hepatic hemangioma initially managed by surveillance. During her observation period, she developed weight loss, constipation, and pancytopenia concerning for Kasabach-Merritt Syndrome. Resection of the hemangioma was complicated by its large size (28.0 × 18.0 × 11.4 cm). Patients with rapidly growing giant liver hemangiomas, even when asymptomatic, should be promptly referred to specialized surgical centers for evaluation and management.

## 1. Introduction

Liver hemangiomas are the most common, benign mesenchymal hepatic lesions occurring in up to 20% of the population. Seldom, giant hemangiomas may cause abdominal pain, congestive heart failure, or even Kasabach-Merritt Syndrome (KMS), a rare life-threatening consumptive coagulopathy. We present the case of a young patient with a rapidly enlarging giant hepatic hemangioma that leads to multiple medical comorbidities. We emphasize the need for recognizing early signs to abandon standard surveillance therapy in patients with rapidly enlarging giant liver hemangiomas and urge for timely referral to specialized hepatobiliary surgical units and surgical resection.

## 2. Case Report

A 37-year-old female with a known history of giant hepatic hemangioma was referred with one-year history of increasing abdominal pain associated with progressive nausea, 60 lbs weight loss and constipation. The hemangioma was initially diagnosed during a workup for lower back pain two years before the onset of abdominal pain. General surgical consultations were sought, and expectant management was advised. The patient had subsequently been treated conservatively for lumbar disk herniation and newly diagnosed irritable bowel syndrome. In our clinic, she appeared pale and malnourished with lumbar lordosis. The abdominal exam revealed generalized distention with a palpable liver edge below the right subcostal margin.

**Figure 1:**
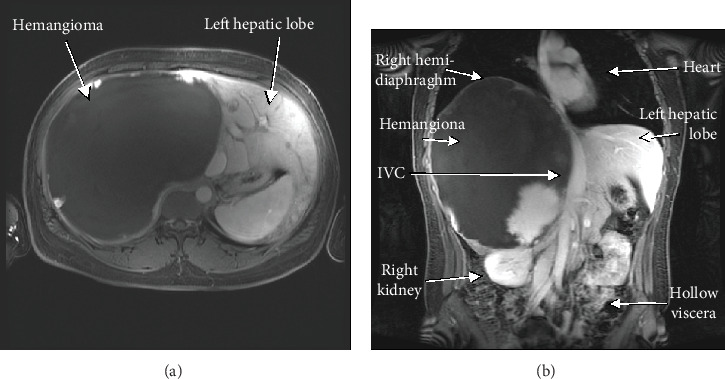
Magnetic resonance imaging of the abdomen. (a) Axial image showing massive T2 hyperintense lesion occupying a majority of the right hepatic lobe. (b) Coronal section showing the giant hemangioma displacing the hepatic hilum, left hepatic lobe, inferior vena cava, right kidney, right hemi-diaphragm, heart, and all the hollow viscera.

Magnetic resonance imaging (MRI) of the abdomen revealed 18.5 (AP) × 19.2 (TV) × 25.3 (SI) cm mass located in the right posterior hepatic lobe consistent with giant hemangioma (Figure 1). A review of radiological records indicated the hemangioma had grown steadily in a diameter from 13 cm in 2015 to 25 cm in 2018. Laboratory evaluation was significant for pancytopenia (Table 1).

Consent for surgical resection was obtained. A surgical set-up has been described earlier. [1] A midline incision with right subcostal extension was placed. The hemangioma arose from the right posterior lobe and was compressing the remaining liver and surrounding structures. Due to tumor size, the right lobe could not be mobilized and access to the vena cava was severely limited. It was therefore decided to proceed with “anterior approach” and resect the mass by creating a plane between the displaced right hepatic vein and the hemangioma. Monopolar coagulation and Cavitron Ultrasonic Surgical Aspirator (CUSA, ValleyLab, USA) were then used to transect the liver parenchyma just lateral to the right hepatic vein. Blood loss was limited through intermittent use of the Pringle maneuver in addition to low central venous pressure anesthesia. The mass was removed uneventfully with an estimated blood loss of 1,200 mL. The patient received two units of packed red blood cells, one of platelets, two of cryoglobulin, and one of fresh frozen plasma. The specimen weighed 3.7 kg (28.0 × 18.0 × 11.4 cm), and histopathologic assessment confirmed cavernous hemangioma.

**Figure 2:**
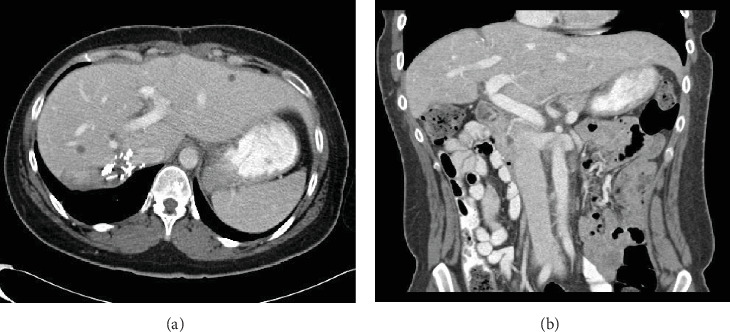
Computed tomography of the abdomen 6 months following giant hemangioma resection. (a) Axial image showing normal residual liver with surgical clips along the edge of inferior vena cava (IVC). (b) Coronal imaging showing resolution of prior displacement of abdominal viscera and IVC.

The patient was discharged home on postsurgical day five. At her six-month follow-up, she had resolution of her abdominal pain and nausea and reported improved bowel function. Her weight loss stabilized. She continued to have mild lower back pain from her lumbar disk herniation. There was interval improvement of her pancytopenia (Table 1). Computed tomography (CT) imaging of the abdomen at 6 months postoperatively is shown in Figure 2.

## 3. Discussion

Hemangiomas are the most common benign hepatic lesion and occur in up to 20% of the population. The classification of hemangiomas based on size varies widely, with giant hemangiomas being described as >5 or even >10 cm [2]. Extremely giant hemangiomas > 20 cm occur less frequently and mainly are discussed in case reports or short reviews detailing the method of resection or rare associated medical sequela [3–5]. The largest such review was performed by Liu et al. and included 36 patients with hemangiomas > 20 cm [3].

Current guidelines advocate for the conservative management of asymptomatic giant liver hemangiomas via serial imaging to monitor growth [6, 7]. Resection is reserved for symptomatic disease, which is considered pain, extrinsic compression, or gastrointestinal issues, with pain being the most commonly cited indication [8–10]. Currently, there is no recommendation to resect hemangiomas for growth alone given possible operative risks. We present a case of a young patient with an enlarging, initially asymptomatic, extremely giant hepatic hemangioma who would have benefitted from surgical resection prior to the development of typical symptoms.

When her hemangioma was initially diagnosed, our patient did not have typical indications for resection such as abdominal pain or extrinsic visceral compression. Over the next three years, surveillance MRIs demonstrated the hemangioma nearly doubling in diameter from 13 cm to 25 cm, with an approximate eight-time increase in absolute volume. During this observation phase, she developed typical compressive symptoms attributable to her hemangioma including nausea, constipation, 60 lbs (27 kg) weight loss, and abdominal pain and was beginning to exhibit early signs concerning for Kasabach-Merritt Syndrome. Surgical resection required large open procedure and, although without complication, was technically difficult given anatomic distortion from tumor size and inability to secure the retrohepatic inferior vena cava. This raises the question of whether surgical resection should be preemptively performed in similar patients who demonstrate persistent hemangioma growth.

While hemangiomas may regress in size over time, more than half of all hemangiomas grow on follow-up imaging. In addition, a larger hemangioma size on initial diagnosis correlated with a higher subsequent rate of growth, with younger patients more likely to have the highest growth rate [11]. Liu et al. found hemangioma growth to be most rapid between the ages of 30-39 years, averaging at 4 cm per year [12]. Our 37-year-old patient showed similar growth with the hemangioma enlarging 12 cm in diameter over the course of three years.

As they grow in size, giant liver hemangiomas are associated with increasing perioperative risk. In a retrospective review, Wahab et al. found that patients undergoing resection for hemangiomas larger than 10 cm had significantly increased intraoperative blood loss with need for blood transfusions and longer operative times [10]. Patients with extremely giant liver hemangiomas (>20 cm) have a significantly increased incidence of preoperative anemia, thrombocytopenia, Kasabach-Merritt Syndrome, and compression of surrounding major vasculature, thus making them higher operative risk candidates [3]. Our unpublished observations support these findings, and we, therefore, recommend early surgical intervention for enlarging giant liver hemangiomas.

We conclude that progressively enlarging hepatic hemangiomas may benefit from surgical resection prior to presentation of typical symptoms. We propose that incidentally discovered giant hemangiomas should be referred to specialized surgical centers for continued follow-up. Early surgical resection (or enucleation) should be considered for giant hemangiomas before anatomic distortion of liver parenchyma or the onset of medical comorbidities make surgical intervention prohibitively risky.

## Figures and Tables

**Table 1:** Perioperative laboratory parameters.

Laboratory value	Reference range	1-month pre-op	Day of surgery	2-week post-op	6-month post-op
*Liver function tests*					
S. aspartate aminotransferase (unit/L)	0–50	23	—	26	22
S. alanine aminotransferase (unit/L)	0–50	11	—	15	18
S. alkaline phosphatase (unit/L)	0–120	60	—	66	71
S. bilirubin, total (mg/dL)	0–1.3	0.7	—	0.7	0.7
S. bilirubin, conjugated (mg/dL)	0–0.4	0.3	—	0.3	0.3
S. protein, total (g/dL)	6.4–8.5	7.3	—	7.0	8.1
S. albumin (g/dL)	3.7–5.2	4.5	—	4.0	4.4
*Hematology*					
White cell count (10^9^ cells/L)	3.9–11.7	3.7	2.7	8.3	4.5
Hemoglobin (g/dL)	12.0–15.0	7.8	6.5	9.9	10.4
Hematocrit (%)	34.8–45.0	25.9	21.1	32	34.5
Platelets (10^9^ cells/L)	172–440	99	91	216	110
*Chemistry*					
S. creatinine (mg/dL)	0.5–1.0	0.8	—	0.9	1.08
*Coagulation*					
Prothrombin time (seconds)	12.3–14.8	19	19.8	17.1	16.6
Activated prothrombin time (seconds)	25–36	—	40	—	—
International normalized ratio	1	1.6	1.6	1.4	1.3
